# Redox Homeostasis and Inflammation Responses to Training in Adolescent Athletes: a Systematic Review and Meta-analysis

**DOI:** 10.1186/s40798-020-00262-x

**Published:** 2020-08-03

**Authors:** Evdokia Varamenti, David Tod, Samuel A. Pullinger

**Affiliations:** 1 Aspire Academy for Sports Excellence, Sports Science Departement, PO Box: 22287, Doha, Qatar; 2 Research Institute for Sport and Exercise Sciences, Liverpool John Moores University, Liverpool, UK

**Keywords:** Acute and chronic responses, Redox homeostasis, Inflammation, Adolescent athletes

## Abstract

**Background:**

Several studies have highlighted the substantial role of the athlete’s redox and inflammation status during the training process. However, many factors such as differences in testing protocols, assays, sample sizes, and fitness levels of the population are affecting findings and the understanding regarding how exercise affects related biomarkers in adolescent athletes.

**Objectives:**

To search redox homeostasis variables’ and inflammatory mediators’ responses in juvenile athletes following short- or long-term training periods and examine the effect size of those variations to training paradigms.

**Methods:**

A PRISMA-compliant systematic review and meta-analysis were conducted. The entire content of PubMed (MEDLINE), Scopus, and Science Direct were systematically searched until December 2019. Studies with outcomes including (1) a group of adolescent athletes from any individual or team sport, (2) the assessment of redox and/or inflammatory markers after a short- (training session or performance testing) or longer training period, and (3) variables measured in blood were retained. The literature search initially identified 346 potentially relevant records, of which 36 studies met the inclusion criteria for the qualitative synthesis. From those articles, 27 were included in the quantitative analysis (meta-analysis) as their results could be converted into common units.

**Results:**

Following a short training session or performance test, an extremely large increase in protein carbonyls (PC) (ES 4.164; 95% CI 1.716 to 6.613; *Z* = 3.333, *p* = 0.001), a large increase in thiobarbituric acid reactive substances (TBARS) (ES 1.317; 95% CI 0.522 to 2.112; *Z* = 3.247, *p* = 0.001), a large decrease in glutathione (GSH) (ES − 1.701; 95% CI − 2.698 to − 0.705; *Z* = − 3.347, *p* = 0.001), and a moderate increase of total antioxidant capacity (TAC) level (ES 1.057; 95% CI − 0.044 to 2.158; *Z* = 1.882, *p* = 0.060) were observed. Following more extended training periods, GSH showed moderate increases (ES 1.131; 95% CI 0.350 to 1.913; *Z* = 2.839, *p* = 0.005) while TBARS displayed a small decrease (ES 0.568; 95% CI − 0.062 to 1.197; *Z* = 1.768, *p* = 0.077). Regarding cytokines, a very large and large increase were observed in IL-6 (ES 2.291; 95% CI 1.082 to 3.501; *Z* = 3.713, *p* = 0.000) and IL-1 receptor antagonist (ra) (ES 1.599; 95% CI 0.347 to 2.851; *Z* = 2.503, *p* = 0.012), respectively, following short-duration training modalities in juvenile athletes.

**Conclusions:**

The results showed significant alterations in oxidative stress and cytokine levels after acute exercise, ranging from moderate to extremely large. In contrast, the variations after chronic exercise ranged from trivial to moderate. However, the observed publication bias and high heterogeneity in specific meta-analysis advocate the need for further exploration and consistency when we deal with the assessed variables to ascertain the implications of structured training regimes on measured variables in order to develop guidelines for training, nutritional advice, and wellbeing in young athletes.

**Trial Registration:**

PROSPERO CRD42020152105

## Key Points

- Significant alterations in oxidative stress and cytokines levels are noted after acute exercise, while the variations after chronic exercise ranged from trivial to moderate.
- The knowledge of the magnitude of those changes could lead to remedies to monitor training, recovery process, nutrition, and athlete’s wellbeing.
- More randomised control studies (RCTs) and improved study design are primarily recommended to strengthen the quality assessment of relevant investigations and reduce the risk of publication bias and high heterogeneity.

## Introduction

Redox homeostasis occurs when a balance between the body’s oxidants and antioxidants exists. On the contrary, an oxidative stress state can indicate a considerable increment of oxidative products or deterioration of various antioxidants to counter the induced stress [1–3].

Sports training can lead to significant alterations in variables related to the athlete’s redox and inflammation status [4, 5]. Through sports practice, athletes are regularly exposed to different types of stress to achieve the desired adaptations, which in turn contribute to performance enhancements. Recently, our knowledge about the role of reactive oxygen and nitrogen species (RONS) on exercise has expanded. It is now well accepted that training adaptations might also be linked to improvements in metabolic changes influenced by exercise-induced oxidative stress [6–8]. Moreover, exercise-induced oxidative stress might display the necessary signalling to determine adaptations to endurance training [6]. Also, initial levels of specific enzymatic antioxidant, like superoxide dismutase (SOD), could be used to predict the capacity of the antioxidant defence system and differentiate between well-trained subjects and controls [9]. It seems that acute alterations which take place during exercise are possibly part of the body’s adaptations mechanism while increases in the level of reactive species or oxidant biomarkers following a more extended period of training may suggest the need for remedies in training plans and nutrition [5].

Well-structured training programmes, which are characterised by a distinct sequence of exercises, interrupt the redox equilibrium, provoking adaptations so that the body can deal with equal loads of RONS in the ensuing practices [10]. Accordingly, our insight of exercise-induced oxidative stress could likewise be relevant for exercise prescription. Hence, when coaches and sports experts aim to enhance the athletes’ performance and recovery processes, variables associated with oxidative stress should be taken into their consideration in order to optimise training load patterns and nutritional interventions, to maximise adaptations and as a result performance, especially in endurance events.

Training activities involving endurance, sprint, or resistance training are likely to induce metabolic stress as well as patterns of muscle damage. Furthermore, exercise-induced muscle damage (EIMD) is linked with oxidative stress through the activity of neutrophils and macrophages [8, 11]. After an injury or muscle impairment, these specific leukocyte subsets infiltrate a damaged tissue for healing by releasing reactive and nitrogen oxygen species (RONS) and by synthesising pro-inflammatory cytokines. During this action, neutrophils can further increase oxidative stress through the “respiratory burst” [8] while nitric oxide (NO) can provoke highly reactive species [8, 10]. Besides, cytokines such as IL-6, IL-1b, and IL-1 receptor antagonists (ra) play a fundamental role in the regulation of inflammation as they contribute to the clearing of antigens and the restoration of tissue [12].

Training can have positive or negative effects on oxidative stress and inflammation variables depending on the training load, the exercises prescribed, the specificity, the athlete’s age, and their training experience [13]. Although distinct types of exercise may provoke different levels of oxidative stress in a specific pattern and lead to inflammatory responses in adolescent athletes, aggregated data related to those responses are elusive. This limitation occurs because different biomarkers, types of specimen, methodologies, sampling times, and testing protocols have been implemented to monitor the induced alterations [5]. Routinely in sports populations, redox homeostasis variations are determined by analysing several biomarkers, mainly in the blood by means of venepuncture sample collections. Such biomarkers can denote a specific type of harm on lipids, proteins, and DNA or the accumulation of certain antioxidants and reactive species. Still, for any distinct impairment that can be caused by a direct attack of reactive species during oxidative stress, varied biomarkers can be used. For example, for the monitoring of lipid peroxidation, several biomarkers (i.e. malondialdehyde (MDA), thiobarbituric acid reactive substances (TBARS), lipid hydroperoxides (LOOHs), conjugated dienes, F_2_-isoprostanes) can be utilised [13, 14]. However, all battery of assays representing a type of damage, including lipid peroxidation, have limitations as a wide range of products are formed in variable amounts, so attention is needed for the choice of the appropriate biomarker and the interpretation of results [2].

With more young athletes engaged in supervised training sessions, it is essential to define the most relevant variables to quantify their training adaptations and responses to different exercise regimes. Thus, this work aimed to (1) systematically search data reporting variations in variables related to redox and inflammation status in adolescent athletes, following short- or prolonged training periods in order for individuals that have an interest in the field gain a better understanding linked to each variable, and (2) examine the effect size of acute and chronic responses to training and identify the most sensitive variables to display those changes.

## Methods

The present systematic review (SR) and meta-analysis (MA) was registered with the PROSPERO database of reviews (registration number: CRD42020152105) and follows the Preferred Reporting Items for Systematic Reviews and Meta-Analyses (PRISMA) statement guidelines [15]. The PRISMA checklist is presented in Additional file 1: Appendix 1, indicating the page numbers where items of information are present in the current manuscript. Also, 20 authors whose papers included in MA (resulting in 27 articles) were contacted by email, of which ten replied and were able to provide data. Seven other articles included full data, and for three studies, results were estimated by using the above-mentioned software.

### Screening—Eligibility

The inclusion criteria were based on the Cochrane guidelines for conducting systematic reviews [16]. The criteria for inclusion and exclusion were set and agreed by all three authors. Following the initial selection process of studies, three authors (EV, SP, and DT) independently completed the eligibility assessment in a blinded standardised way by screening the titles and abstracts. To be considered eligible, the manuscript had to meet the following inclusion criteria:

1. Language—published in English in a peer-reviewed journal.
2. Population—healthy adolescent participants with a mean age ranging from 12 to 18 years of age.
3. Training period—short-term training (i.e. a single training session or performance testing session) or long-term training periods (i.e. a more extensive period of training involving a micro-, meso-, and macro-cycle ranging from 45 days to 1 year).
4. Biomarkers—biomarkers associated with redox status and inflammation measured in blood (see the “Study Selection—Key Redox Status and Inflammation Mediator Variables” section).
5. Design—non-randomized control trials (NRCTs) and case-control study designs.

### Literature Search Strategy and Information Sources

**Fig. 1:**
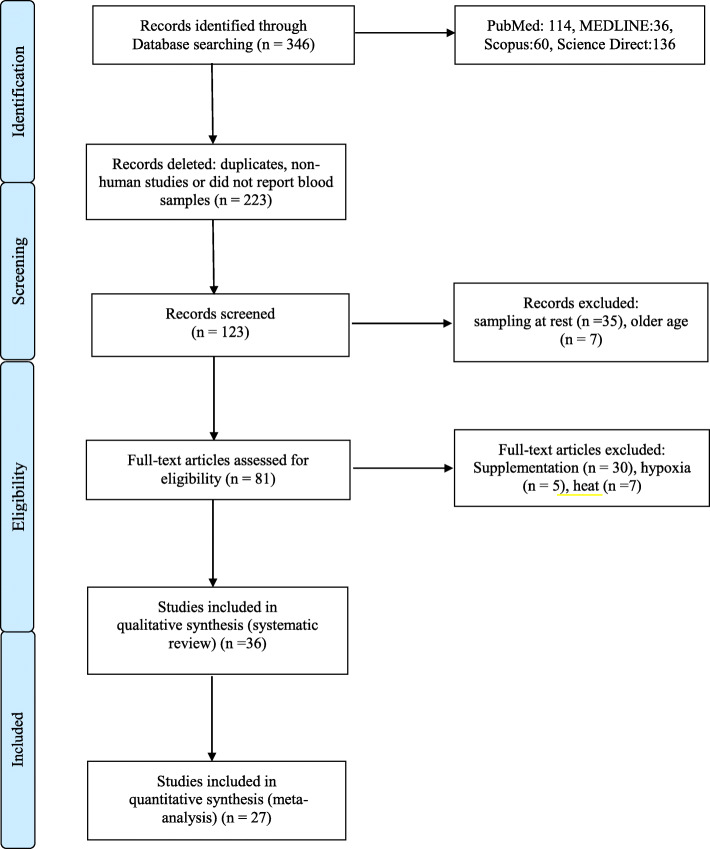
The PRISMA flowchart showing the flow of papers through the study selection process

A computerised English-language literature search of electronic databases: PubMed (MEDLINE), Scopus, and ScienceDirect was conducted (December 2019). A search for relevant content related to fluctuations in biomarkers associated to oxidative stress and inflammation mediators in young athletic populations using the following search syntax was performed: (“oxidative stress” OR “oxidative damage” OR “redox alterations” OR “redox status”) AND (“adolescent athletes” OR “young athletes”) AND (“post-training” OR “post-exercise”) or (“proinflammatory cytokines” OR “inflammation” OR “IL-6” OR “IL-1ra”) AND (“adolescent athletes” OR “young athletes”) AND (“post-training” OR “post-exercise”). The initial search was conducted by one author (EV). The search syntax was combined with Boolean operators, and the quotation marks were used for phrase searching (i.e. combinations of two or more words). The search was limited to papers published in the English language, which included the monitoring of the studied parameters following a short- or a long-term period of training. In addition, the reference lists of articles retrieved were screened manually for additional relevant papers, as part of the secondary search to uncover any additional articles that met the inclusion criteria. Two authors (SP and DT) independently carried out the searches for study selection to minimise potential selection bias. Disagreements were discussed between three authors (EV, SP, and DT) and resolved by consensus. Figure 1 presents the PRISMA flowchart showing the flow of papers through the study selection process.

### Study Selection—Key Redox Status and Inflammation Mediator Variables

The current systematic review focused on exploring (i) endogenous antioxidant variables: superoxide dismutase (SOD), glutathione peroxidase (GPx), and catalase (CAT); (ii) oxidative stress markers: thiobarbituric acid reactive substances (TBARS), malondialdehyde (MDA), reduced glutathione (GSH), protein carbonyls (PC), and total antioxidant capacity (TAC); and (iii) inflammatory mediators: interleukin-6 (IL-6), interleukin-1receptor antagonist (IL-1ra), tumour necrosis factor-α (TNF-α), and interleukin-10 (IL-10). In instances where the title and abstract did not contain enough detail to indicate whether an article was relevant to the review, the complete article was obtained and read. This decision enabled the authors to determine whether the paper met the primary inclusion criteria. In instances where the primary purpose of the article was not an investigation exploring alterations in biomarkers associated with oxidative stress and inflammation due to applied training programs, the papers were excluded from the review. Specifically, studies involving supplementation interventions, exercise protocols in hypoxia or heat, and specific medical conditions (e.g. asthma) were excluded a priori, as they could have affected outcomes in those studies. Letters to the editor and conference abstracts were also excluded as these studies were not found to be methodologically quality assessable and/or critically appraisable.

### Data Extraction

Data extraction was performed by one author (EV) independently and a data check performed by a second author (SP), finding a 100% data check agreement. The following data were extracted from the included studies: (1) the study authors and date, (2) the number of participants and their characteristics (e.g. sample size, sex, sport, athletes’ features), (3) the explored redox status and inflammation variables, (4) the sampling time and description of activity/performance test used (e.g. training phase, pre- vs post-exercise, time of sampling), and (5) effects of applied testing protocols or training modalities on examined biomarkers.

### Assessment of Methodological Quality

Methodological quality assessment was conducted on each included article using a modified Downs and Black scale [17], which is appropriate for non-randomized control trials (NRCTs) and case-control study designs [18]. The Methodological Quality Checklist for each study includes twenty-seven items. For this review, twenty-six questions were scored totalling up to 27 possible points, as has previously been done [18]. The questions were categorised under 4 sections: reporting (10 items; items 1–10), external validity (3 items; items 11–13), internal validity—study bias (7 items; items 14–20), and internal validity—confounding selection bias (6 items; items 21–26). The quality assessment of the articles was conducted by two reviewers (SP and EV). The observed differences were resolved by a third reviewer (DT), and details for overall study scores are provided in Additional file 1: Appendix 2.

### Statistical Analysis

#### Assessment of Effect Size

Statistical analyses were performed using “The Comprehensive Meta-analysis software” (version 3.0, Biostat Inc., Englewood, NJ, USA). In cases of missing data, authors were contacted via email and asked to provide the necessary information. If no response was received, possible means and SDs were estimated from figures using computer software (ImageJ, Bethesda, MD, USA; Imagej.net). All analyses were conducted using a random-effects model to account for measurement variability and heterogeneity among the studies. For the completion of meta-analysis for each examined variable, a minimum of five studies was agreed. Further, the effect size (ES) was calculated according to Hedges’ *g* [19], which is similar to Cohen’s *d*, but it includes an adjustment for small sample size. The magnitude of the effects sizes was interpreted as changes using the following criteria: trivial (< 0.20), small (0.21–0.60), moderate (0.61–1.20), large (1.21–2.00), very large (2.01–4.00), and extremely large (> 4.00) [20]. The mean differences and 95% confidence intervals (CIs) were calculated for the included studies.

### Assessment of Heterogeneity

The chi-square Cochran’s *Q* test (*χ*^2^) is meant to test the null hypothesis that there is no dispersion across effect sizes. As we wish also to quantify this dispersion, the measure of *I*-squared (*I*^2^) and tau-squared (*t*^2^) were implemented. Therefore, the *I*^*2*^ statistic was used for the evaluation of statistical heterogeneity among studies while values of 25, 50, and 75% represent low, moderate, and high statistical heterogeneity, respectively [21]. Furthermore, the tau-squared (*t*^2^) estimates the “between studies” variance [13, 21].

### Publication Bias

The publication bias was assessed by examining the asymmetry of the funnel plots using Egger’s test, and significant publication bias was considered when *p* > 0.10 [22]. Whether publication bias was recorded, then, Duval and Tweedie’s “Trim and Fill” method was further considered which not only indicates the significance of publication bias but also provides bias-adjusted results [23].

## Results

### Literature Search Results

The literature search ended in September 2019, and the primary database search revealed 346 potentially relevant journal articles. Figure 1 presents the number of articles found in each electronic database and a detailed PRISMA flow chart of the literature search, including all the steps performed. Once duplicates and non-relevant papers were removed, 123 titles and abstracts remained in the reference manager (EndNote V.X.8). Following the examination of titles, abstracts, and keywords of all these manuscripts, 81 academic studies were deemed eligible and retained for full-text analysis. After additional full-text analysis, 36 studies were deemed eligible and included in the systematic review. A total of 27 studies were used to conduct the meta-analysis. Concerning the meta-analyses related to redox homeostasis variables, studies with male adolescents and with results collected immediately post-training or performance testing that could be converted in similar units were considered. The reason for focusing on male adolescents is that many physical and physiological changes occur between 12 and 14 years of age, especially in girls, which could have an impact on the results of this systematic review and meta-analysis. From the included studies, 5 have a mean age for young athletes below 14 years [16, 24–27]. Moreover, twenty-two out of the 27 included studies are characterised as uncontrolled trials (UCTs) [9, 16, 24, 25, 28–45] and five studies as control trials (CTs) [26, 27, 46–48].

### Data Extraction

A summary of the studies included in the systematic review is presented in Tables 1, 2, and 3. In total, 856 adolescents including 569 males, 174 females, and 113 male controls were included ranging from various sports (e.g. athletics, basketball, cross-country, handball, judo, multi-sport, rowing, soccer, swimming, volleyball, water polo, tennis, and wrestling). Table 1 displays the twenty-one studies where the effect of a short-term training period, event, or performance testing on redox status variables was examined. In Table 2, the 8 studies with an extended period of training are shown, of which 3 studies include short and long protocols [26, 45, 53]. In Table 3, the ten studies which discussed results relevant to cytokines are presented. For the redox status meta-analyses, a total of 459 young male athletes were included from 9 different sports (athletics, basketball, handball, multi-sport, rowing, soccer, swimming, volleyball, and wrestling).

**Table 1:** Summary of the acute responses of redox homeostasis variables in adolescent athletes with an overview of the variables examined, the type of activity and sampling times, and the main findings in relation to each variable

Author and date, sport	Participants	Redox status variables examined	Type of activity and sampling times	Main findings
da Costa et al. 2011 [49] , soccer	10 trained male soccer players, age 18.3 ± 0.7 years	TPAP, MDA, CK, LOOH	Blood samples before, during, and after a Loughborough Intermittent Shuttle Test (LIST)	Immediately after the test athletes had: ↓TPAP, ↑MDA, CK, ↔LOOH
Dane et al. 2008, [46], athletics	19 controls, age 18.8 ± 1.9 years, and 20 male runners, age 18.6 ± 2.0 years	MDA, GPx, SOD	Pre- and post-running on a treadmill	Runners after test had: ↔MDA, GPx, SOD
Djordjevic et al. 2010 [9], handball	24 male young handball players, age 16.1 ± 0.6 years	O_2_^−^, H_2_O_2,_ GSH, CAT, TBARS, SOD, NO_2_	Blood samples pre- and post a graded exercise test (GXT) on a bicycle ergometer	Post-test athletes had: ↑TBARS, NO_2,_ O_2_^−^, H_2_O_2_ ↓SOD, CAT, GSH
Djordjevic et al. 2012 [47], handball	58 adolescent males and 19 control, age 16–19 years	O_2_^−^, H_2_O_2,_ NO_2_, SOD, TBARS, CAT, GSH	Blood samples pre- and post of a graded exercise test (GXT) on a bicycle	At rest athletes displayed: ↑NO_2_, SOD, CAT, GSH, ↓TBARS, ↔ O_2_^−^, H_2_O_2_. Post-test athletes had: ↓O, ↔TBARS, CAT, GSH, SOD, NO_2_^−^, H_2_O_2_
Escobar et al. 2009 [30], soccer	18 adolescent males, age 17.0 ± 0.5 years	SOD, CAT TBARS, PC	Pre- and post-10× (2 × 200 m) sprints with 30 s rest between each 200 and a 90-s rest after two consecutive sprints	After the test, athletes had: ↑TBARS, PC, SOD, CAT
Hammouda et al. 2012 [31], soccer	18 male football players, age 17.5 ± 0.4 years	Bilirubin, UA, TAS	Blood samples were collected 5 min before and 3 min after a 30 s Wingate test	After exercise athletes had: ↑UA, TBIL, TAS
Hamurcu et al. 2010 [50], wrestling	18 adolescent males and 18 controls, age 13–15 years	8-OHdG, NO, PON1	Pre- and post of 1.5 h of wrestling exercise	After test wrestlers had: ↑NO, ↔PON1, 8-OHdG
Inal et al. 2000 [32], swimming	5 males and 4 females and 6 males and 10 females for 100 and 800 m swim, age 15–21 years	GSH, GPx, CAT	Measures pre-, post-, 20 min post, and 40 min post 100 m and 800 m swimming tests	After both swim tests, athletes had: ↑GPx, CAT, ↓GSH
Kabasakalis et al. 2014 [33], swimming	15 adolescent males and 15 females, age 14–18 years	8-OHdG, MDA, PC, GSH, UA, bilirubin	Pre-, post-, 1 h, 24 h post-2000 m continuous and 6 × 50 m intermittent maximal swim	Male swimmers had: ↑MDA both trials, ↓GSH 24 h post both trials, ↑UA, bilirubin 24 h intermittent test, ↔ PC
Kabasakalis et al. 2019 [51], swimming	16 males, age 15.9 ± 1.0 years, and 16 female swimmers, age 15.4 ± 1.0 years	Irisin, GSH, TAC, 8-OHdG	Pre- and post of two sets of 4 × 50 m maximal freestyle swimming separated by 10 min	In pooled males and females: ↑GSH, TAC at post 1 and at post 2 trials
Kyparos et al. 2009 [35], rowing	19 international level rowers, 18.5 ± 0.9 years	GSH, GSSG, TAC, CAT, TBARS, PC	Blood samples pre- and post a simulated 2000 m rowing race, on a rowing ergometer	After test rowers had: ↓GSH/GSSG ↔GSH, ↑GSSG, TBARS, PC, TAC, CAT
Kurkcu et al., 2010 [34], basketball	7 adolescent females and 55 male adolescents, age 15.3 ± 1.8 years	TAS, OSI, TOS, LOOH	Pre- and post a circuit exercise program	After the exercise program, athletes had: ↑TOS, OSI, ↓TAS, ↔LOOH
Maric 2018 [52], basketball	12 female athletes, age 17.8 ± 4.1 years	TBARS, GSH, CAT, SOD, O_2_^−^, H_2_O_2_, NO	Pre- and post a continuous or HIIT aerobic training (high-intensity interval training)	After continuous: ↓NO, TBARS, CAT. After HIIT: NO, ↑CAT
Nikolaidis 2007 [24], swimming	11 adolescent males and 11 females, age 11–13 years	GSH, GSSG, TBARS, PC, TAC	Pre- and post of 12 bouts of 50 m swimming at 70–75 %, with 1 min rest periods	Post-test, in both genders: ↓GSH, GSH: GSSG, ↑GSSG, TBARS, CAT, PC, TAC
Otocka-Kmiecik et al. 2010 [42], n/a	32 physically active adolescents, age 18.4 ± 2.6 years	PON, FRAP, ARE, UA, bilirubin, TBARS	All participants completed a maximal exercise on a treadmill	After the test sportsmen had: ↑UA, FRAP, ↔ PON, ARE, bilirubin
Otocka-Kmiecik et al. 2014 [48], n/a	26 physically active and 20 sedentary adolescents, age 17.0 ± 1.0 years	PON, FRAP, UA, bilirubin, TBARS, ARE	Participants performed a maximal exercise on a treadmill	After test physical active adolescents had: ↑UA, FRAP, ↔ PON, ARE, bilirubin
Sopic et al. 2014 [53], soccer	16 soccer players, age 18.1 ± 0.3 years	O_2_^−^, MDA, SH, TAS, TOS, PAB	Pre- and after a single soccer training	After a single training, athletes had: ↓SH-groups, TOS, PAB, ↔O_2_, MDA, TAS
Tauler et al. 2008 [43], swimming	15 adolescent males, mean age of 16 years and 8 females, age of 14.7 years	MDA, CI, various vitamins	Pre- and post a swimming session	After the session, swimmers had: ↔ PC both in boys and in girls, ↑MDA increased in boys
Tian et al. 2010 [44], athletics	12 adolescent males, age 16–17 years	TBARS, GSH, UA, XO, SOD, CAT	Pre-, post-, 2 h- ,4 h-, and 24 h- post of 21 km run	Athletes had 2 h post: ↑UA, CAT; 4 h post: ↑UA, GSH, ↓XO; 24 h post: ↑TBARS, CAT
Tong et al. 2013 [45], athletics	10 adolescent runners, age 15.5 ± 1.3 years	TBARS, XO, CAT, GSH, SOD, T-AOC	Pre- and post a 21-km run, twice a year	At preseason and post 21 km run, athletes had: ↓TBARS, SOD, ↔XO, CAT, T-AOC, GSH
Zalavras et al. 2015 [26], athletics	13 trained (TAD) and 11 untrained (UAD) adolescents, age 14.1 ± 1.1 years and 14.8 ± 0.9 years, respectively	TAC, GSH, CAT, TBARS, PC, UA, bilirubin	Athletes were monitored at pre-, post-, and 1 h post of a maximal test at the beginning of the season, after 6 (mid) and after 11 months (post) of training	After test at pre-season, athletes had: post and 1 h post ↑PC, TBARS, UA, bilirubin, only post ↑CAT, TAC, and ↔GSH

*O*_*2*_^−^ superoxide anion radical, *H*_*2*_*O*_*2*_ hydrogen peroxide, *XO* xanthine oxidase, *NO* nitric oxide, *NO*_*2*_*-* nitrite, *GSH* reduced glutathione, *GSSG* oxidised glutathione, *SH* sulfhydryl groups, *TBARS* thiobarbituric acid reactive substances, *MDA* malondialdehyde, *LOOH* lipid hydroperoxide, *PC* protein carbonyls, *PON* paraoxonase activity-1, *CI* carbonyls index, *AOPP* advanced oxidation proteins, *8-OHdG* 8-hydroxy-2′-deoxyguanosine, *SOD* superoxide dismutase, *CAT* catalase, *GPx* glutathione peroxidase, *TAC* total antioxidant capacity, *TAS* total antioxidant status, *TOS* total oxidative status, *T-AOC* total antioxidant capacity, *OSI* oxidative stress index, *PAB* pro-oxidant-antioxidant balance, *ARE* arylesterase activity, *FRAP* ferric reducing ability of plasma, *UA* uric acid, *TPAP* total plasma antioxidant potential, *CK* creatine kinase

**Table 2:** Summary of the chronic responses of redox homeostasis variables in adolescent athletes with an overview of the variables examined, the type of activity and sampling times, and the main findings in relation to each variable

Author and date, sport	Participants	Redox status variables examined	Type of activity and sampling times	Main findings
Kabasakalis et al. 2009 [16], swimming	11 adolescent boys and 13 adolescent girls, age 10–11 years	GSH, GSSG, TAC, CAT, TBARS	Swimmers were monitored pre-, after 13 and 23 weeks of regular swimming period	Post 13 weeks, athletes: ↑ GSH, GSH/GSSG, ↓GSSG, ↔TAC, CAT, TBARS. Post 23 weeks: ↑GSH, ↔TAC, CAT, TBARS
LeMoal et al. 2016 [36], soccer	19 elite professional soccer players, age 18.3 ± 0.6 years	SOD, GPx, GSH/GSSG	The variation of antioxidants was monitored in July, September, December, January, and May	Athletes displayed: ↔ SOD, GPx
Sahin et al. 2013 [25], swimming	10 adolescent males, age 12.7 ± 0.4 years, and 19 females, age 12.1 ± 0.3 years	GSH, NO, TAS, SOD, CAT, GPx, TP-SH, PC, TBARS	Athletes were evaluated at the beginning, after 8 and 16 weeks of a regular swimming period	After 16 weeks athletes had: ↑TBARS, PC, NO, SOD, CAT, GSH ↓GPx, ↔TAS
Sopic et al. 2014 [53], soccer	16 soccer players, age 18.1 ± 0.4 yrs.	O_2_-, MDA, TAS, TOS, PAB, SH	Athletes were assessed after 45 days of preparation	After 45 days of training athletes displayed: ↓TOS, MDA, ↑SH, ↔TAS, PAB, O_2_
Tong et al. 2013 [45], athletics	10 adolescent runners, age 15.5 ± 1.3 years	TBARS, XO, CAT, GSH, SOD, T-AOC	Runners performed a 21-km running race twice; at pre-season and after 1 year of training	At the end season race, athletes displayed post 21-km: ↓TBARS, SOD, ↑XO, CAT, ↔GSH, T-AOC
Vujovic et al. 2013 [54], soccer	12 soccer players, age of 17.3 ± 0.5 years	SOD, SH, O_2_, MDA, AOPP	Soccer athletes were measured before and after 12 weeks of endurance soccer training	After 12 weeks athletes had: ↓SOD, ↑O_2_, ↔MDA, AOPP, SH
Zalavras et al. 2015 [26], athletics	13 trained (TAD) and 11 untrained (UAD) adolescents, age 14.1 ± 1.1 years and 14.8 ± 0.9 years respectively	TAC, GSH, CAT, TBARS, PC, UA, bilirubin	Athletes were monitored at pre-, post-, and 1 h post of a maximal test at the beginning of the season, after 6 (mid), and after 11 months (post) of training	Athletes had: ↑PC, TBARS, UA, TAC post and 1 h post-test at mid- and post-season (except PC 1 h at mid), ↓GSH post-test at post-season and ↑CAT post at mid- and post- season
Zivkovic et al. 2013 [27], soccer	26 adolescent males, age 12–13 years and 26 age-matched controls	TBARS, NO, O_2_^-^, H_2_O_2_, SOD, CAT, GSH	Athletes were monitored pre- and post of a 6-month soccer training	↑TBARS, NO_2_, ↑SOD, CAT, ↔O_2_^–^, H_2_O_2_, ↓GSH

*O*_*2*_^*-*^ superoxide anion radical, *H*_*2*_*O*_*2*_ hydrogen peroxide, *XO* xanthine oxidase, *NO* nitric oxide, *GSH* reduced glutathione, *GSSG* oxidized glutathione, *SH* sulfhydryl-groups, *TBARS* thiobarbituric acid reactive substances, *MDA* malondialdehyde, *LOOH* lipid hydroperoxide, *PC* protein carbonyls, *CI* carbonyls, *AOPP* advanced oxidation proteins, *SOD* superoxide dismutase, *CAT* catalase, *GPx* glutathione peroxidase, *TAC* total antioxidant capacity, *T-AOC* total antioxidant capacity, *TAS* total antioxidant status, *TOS* total oxidative status, *PAB* pro-oxidant-antioxidant balance, *UA* uric acid, *CK* creatine kinase

**Table 3:** Summary of the responses to training on inflammation markers in adolescent athletes with an overview of the variables examined, the type of activity and sampling times, and the main findings in relation to each variable.

Author and date, sport	Participants	Redox status variables examined	Type of activity and sampling times	Main findings
Brunelli et al. 2014 [55] basketball	11 male adolescent athletes, age 13.3 ± 0.6 years	IL-6, IL-10, C-RP, TNF-α	Four blood samples were collected at pre-season, preparatory, and competitive time points	Competitive period compared to preseason: ↑TNF-α, C-RP, ↓IL-10. Competitive period compared to preparatory period: ↑IL-6
Eliakim et al. 2009 [28], volleyball	14 adolescent males, age 16.3 ± 1.1 years, and 13 females, age 16.0 ± 0.4 years	GH, IGF-I, T, C, IL-6, IL-1ra	The effect of a typical volleyball practice. Blood samples collected pre- and post-training	In both genders, after test: ↔IL-1ra, ↑IL-6
Eliakim et al. 2013 [29] volleyball	13 female, national team level players, age 16.0 ± 1.4 years	GH, IGF-I, IGFB3, C, IL-6, IL-1ra	Samples obtained pre-, post-, 1 h post a volley training at preseason and after 7 weeks of preparation	In both conditions, athletes had: ↑IL-6, ↔IL-1ra
Jurimae at al. 2018 [56], rowing	15 female rowers, age 18.3 ± 1.6 years	IL-2, IL-4, IL-6, IL-8, IL-10, TNF-α, IL-1α, IL-1β, VEGF, MCP-1	Blood samples pre- and 1 h post-endurance exercise	↑IL-6, IL-8, VEGF, MCP-1
Meckel et al. 2009 [37], handball	12 junior handball players, age range 17–20 years	GH, IGF, IL-6, IL-1b, IL-1ra, IL-10	Sprint run on a treadmill, at an intensity of 80%. Samples collected at pre-, after each 250-m run, and 60 min post-last 250 m	After exercise athletes displayed: ↑IL-6, ↔ IL-1b, IL-1ra, IL-10
Nemet et al. 2002 [38], wrestling	11 high school boys, aged range 14–18.5 years	IL-6, TNF-α, IGF-1, IGFBP1, IGFBP3, IL-1ra, IL-1b	A single, typical, 1.5 h wrestling practices session. Samples collected pre- and post-training	After typical training wrestlers had: ↑TNF-α, IL-1ra, IL-1b, IL-6
Nemet et al. 2003 [39], water polo	10 elite female water polo players, age 15.1 ± 0.3 years	IL-6, TNF-α, IL-1ra, IL-1b	Pre- and post a typical, 1.5 h water polo practice session	After a water polo session, athlete had: ↑IL-6, IL-1ra, ↔ IL-1b, TNF-α
Nemet et al. 2004 [40], wrestling	11 elite male wrestlers, age 16.5 ± 0.5 years	IL-6, TNF-α, IL-1ra, IL-1b	During a school year, athletes were monitored at pre, mid-, peak-, post- training season.	Athletes displayed at: Mid-season: ↑IL-1ra, IL-6. Post-season: ↓IL-1ra, IL-6. All year: ↔IL-1b, TNF-α
Nemet et al. 2009 [41], cross-country	8 elite female cross-country runners, age 16.8 ± 0.5 years	IL-6, IL-1ra	Pre- and post a typical one-hour endurance training. Blood samples obtained pre- and post-training.	Post training athletes had|: ↑IL-6, IL-1ra, TNF-α, IL-1b
Ziemann et al. 2013 [57], tennis	15 adolescent athletes, age 16 years	H_2_O_2_, IL-1b, TNF-α, IL-6, IL-10, Hsp27, Hsp70, CK	Blood was collected 3 times during a tennis camp: after arrival, after 3 days of active rest and at the end of the camp (14 days).	Post tournament: ↑H_2_O_2_, IL-1b, TNF-α, HSP70. After the conditioning camp: ↓H_2_O_2,_, IL-1b, TNF-α, HSP70, CK, ↑IL-6, IL-10, HSP27

*IL-6* Interleukin-6, *IL-10* interleukin-10, *IL-1ra* interleukin-1 receptor antagonist, *IL-1b* interleukin, *IL-2* interleukin, *IL-4* interleukin, *IL-8* interleukin, *C-RP* C-reactive protein, *TNF-α* tumour necrosis factor-α, *T* testosterone, *C* cortisol, *GH* growth hormone, *IGF-1* insulin-like growth, *IGFB3* insulin-like growth binding protein-3, *VEGF* vascular endothelial growth factor, *MCP-1* monocyte chemoattractant protein-1, *Hsp 27* heat shock protein-27, *Hsp70* heat shock protein-70, *H*_*2*_*O*_*2*_ hydrogen peroxide, *CK c*reatine kinase

### Methodological Quality

The methodological quality scores of the included studies ranged from 16 to 23. Twelve of the included studies [26–29, 37–39, 41, 46–48, 55] were of strong quality (rating above 75%), while the rest of the investigations were deemed moderate (rating 50 to 75%) (Additional file 1: Appendix 2).

### Meta-analysis Findings

**Fig. 2:**
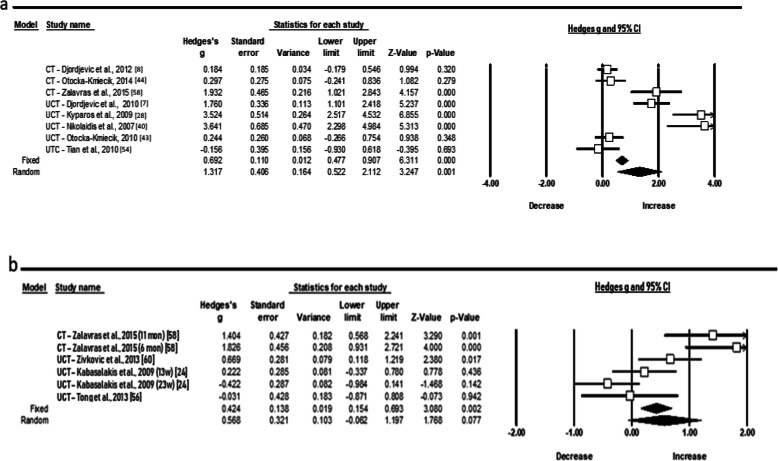
Forest plots demonstrating **a** acute and **b** chronic responses of thiobarbituric acid reactive substances (TBARS) to training in adolescent athletes

In total, ten meta-analyses were conducted, and the significance level of *p* < 0.05 was used for all analyses. Seven out of the 10 performed meta-analyses showed a significant effect size. In particular, the random pooled ES of eight studies included in the meta-analysis showed a large acute increase of TBARS levels (ES 1.317; 95% CI 0.522 to 2.112; *Z* = 3.247, *p* = 0.001) after performing a maximal test by young athletes in several sports (Fig. 2a). The high heterogeneity (*χ*^2^ = 83.3, *p* = 0.000, *I*^2^ = 91.59, *t*^2^ = 1.153) illustrates that 91.59% of the variance reflects differences in the true ES while the other percentage reflects sampling error. On the contrary, the estimated random pooled ES for chronic response of TBARS is small and the statistical heterogeneity low (ES 0.568; 95% CI − 0.061 to 1.197; *Z* = 1.768, *p* = 0.077; *χ*^2^ = 25.7, *p* = 0.000, *I*^2^ = 80.59, *t*^2^ = 0.487) (Fig. 2b). As publication bias was recorded for chronic TBARS responses, the Duval and Tweedie’s “Trim and Fill” method was applied. Consequently, one study trimmed, and the new ES was even lower but still small (i.e. ES 0.334; 95% CI − 0.334 to 1.003).

**Fig. 3:**
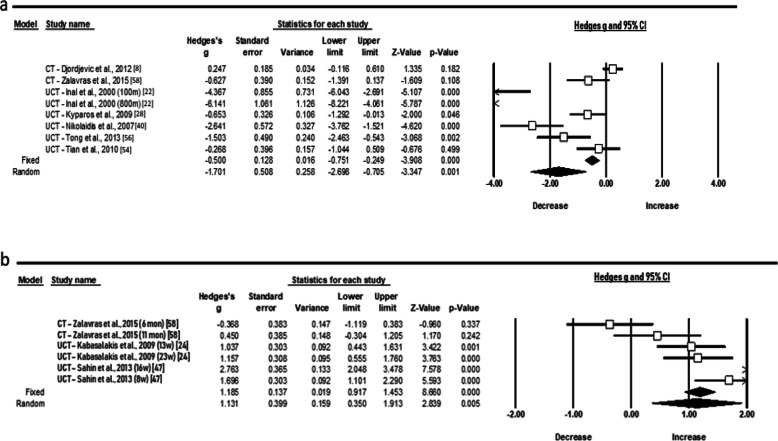
Forest plots demonstrating **a** acute and **b** chronic responses of glutathione (GSH) to training in adolescent athletes

The random pooled ES of eight studies showed a large decrease of GSH level (ES − 1.701; 95% CI − 2.698 to − 0.705; *Z* = − 3.347, *p* = 0.001) immediately after a short duration of training but a moderate increase following an extended period of training in young athletes (ES 1.131; 95% CI 0.350 to 1.913; *Z* = 2.839, *p* = 0.005) (Fig. 3a, b). The statistical heterogeneity was high for both GSH conditions (*χ*^2^ = 83.8, *p* = 0.000, *I*^2^ = 91.65, *t*^2^ = 1.757) and (*χ*^2^ = 41.8, *p* = 0.000, *I*^2^ = 88.05, *t*^2^ = 0.836), respectively. As publication bias was also recorded for chronic GSH responses, the “Trim and Fill” method was applied. However, the ES did not change.

**Fig. 4:**
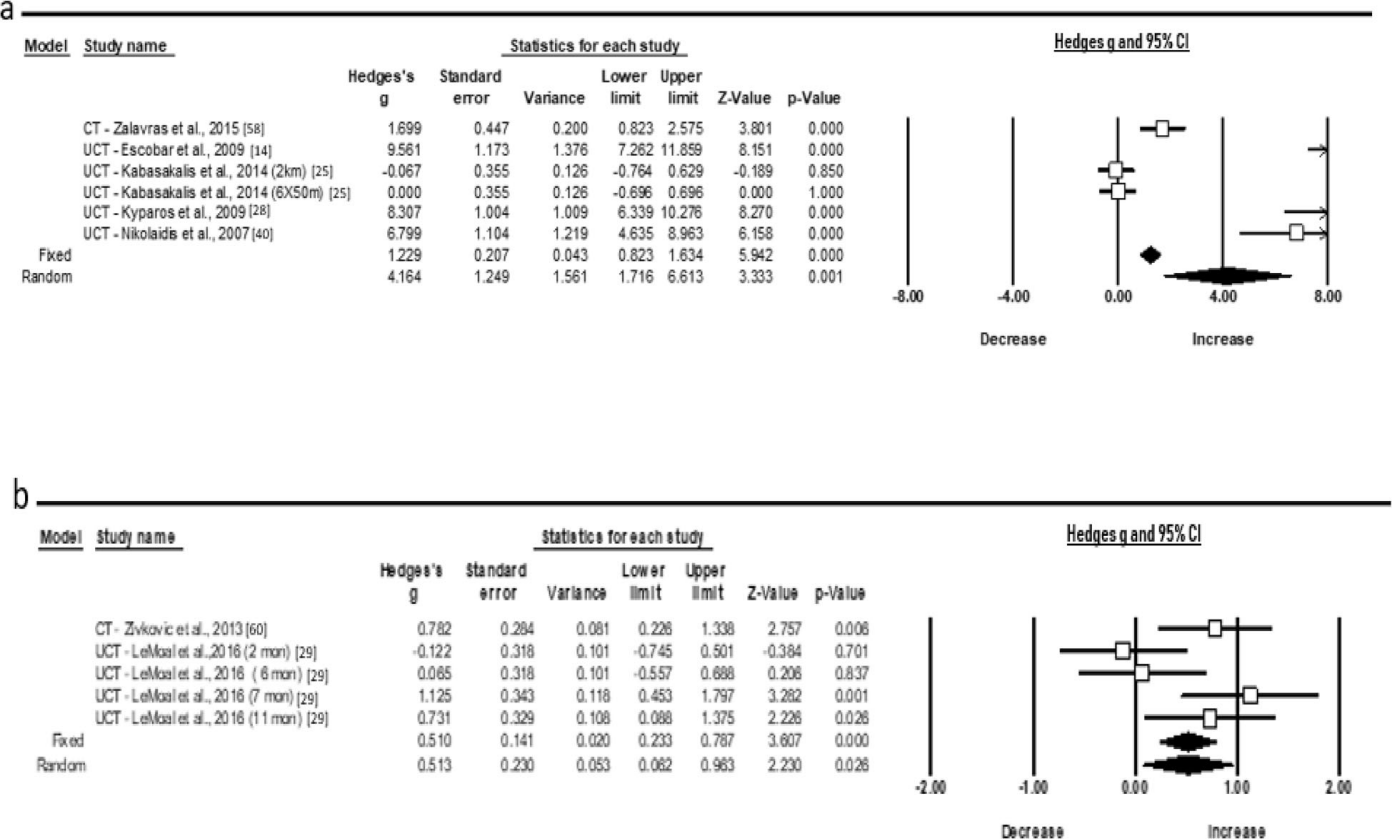
Forest plots demonstrating **a** acute protein carbonyls (PC) and **b** chronic superoxide dismutase (SOD) responses to training in adolescent athletes

Regarding protein carbonyls (PC), an extremely large increase (ES 4.164; 95% CI 1.716 to 6.613; *Z* = 3.333, *p* = 0.001) was observed following an acute period of training (Fig. 4a) with the heterogeneity to be high (*χ*^2^ = 151.9, *p* = 0.000, *I*^2^ = 96.70, *t*^2^ = 8.718). From endogenous enzymes, the random pooled ES of seven studies showed a small increase of SOD level (Fig. 4b) following chronic training (ES 0.513; 95% CI 0.062 to 0.963; *Z* = 2.230, *p* = 0.026). The statistical heterogeneity was high as well (*χ*^2^ = 10.5, *p* = 0.033, *I*^2^ = 61.9, *t*^2^ = 0.163). Despite the recorded publication bias in chronic SOD concentrations, the imputed point estimate did not change after using Trim and Fill adjustment.

**Fig. 5:**
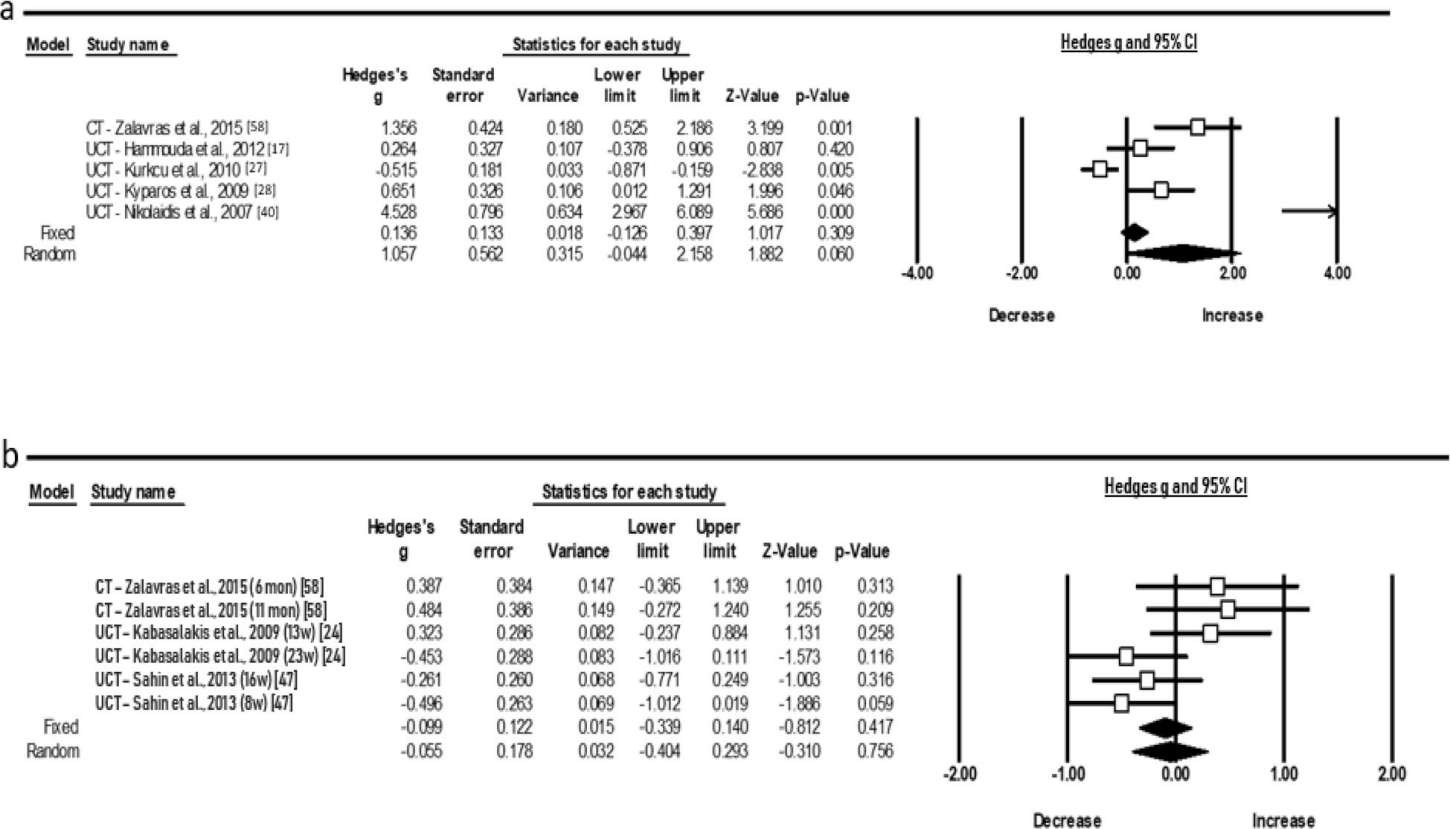
Forest plots demonstrating **a** acute and **b** chronic responses in total antioxidant capacity (TAC) to training in adolescent athletes

**Fig. 6:**
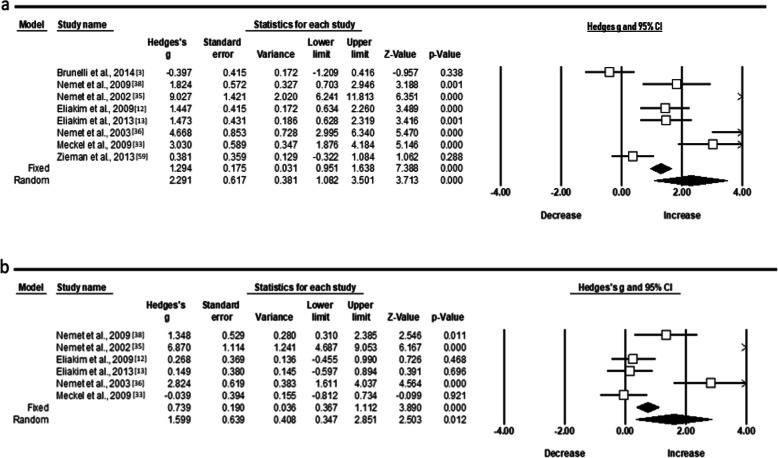
Forest plots demonstrating **a** interleukin IL-6 and **b** IL-1 receptor antagonist (ra) responses to training in adolescent athletes

Further, the random pooled ES of six studies showed a moderate acute increase of TAC level (ES 1.057; 95% CI − 0.044 to 2.158; *Z* = 1.882, *p* = 0.060) and a trivial ES (Fig. 5a, b), after a longer period (ES − 0.055; 95% CI − 0.404 to 0.293; *Z* = − 0.310, *p* = 0.756). The statistical heterogeneity was high for both conditions (*χ*^2^ = 54.2, *p* = 0.001, *I*^2^ = 91.13, *t*^2^ = 1.390) and (*χ*^2^ = 10.2, *p* = 0.068, *I*^2^ = 51.24, *t*^2^ = 1.252), respectively. In relation to cytokines (Fig. 6a, b), the random pooled ES of eight and six studies showed a very large and large increase in IL-6 and IL-1ra (ES 2.291; 95% CI 1.082 to 3.501; *Z* = 3.713, *p* = 0.000 and ES 1.599; 95% CI 0.347 to 2.851; *Z* = 2.503, *p* = 0.012) and high heterogeneity following acute exercise modality, correspondingly (*χ*^2^ = 78.2, *p* = 0.000, *I*^2^ = 91.04, *t*^2^ = 2.621 and *χ*^2^ = 45.8, *p* = 0.000, *I*^2^ = 91.27, *t*^2^ = 2.107).

For the examination of publication bias, Egger’s test [22] was performed for each group of studies to provide statistical evidence of funnel plot asymmetry (Table 4). The results indicated publication bias for changes in TBARS, GSH, and SOD after chronic exercise (i.e. *p* > 0.10). For these specific analyses, the “Trim and Fill” method was further performed to provide bias-adjusted results [23].

**Table 4:** Publication bias of included in meta-analysis studies

Statistics	TBARS acute exercise	TBARS chronic exercise	GSH acute exercise	GSH chronic exercise	PC acute exercise	SOD chronic exercise	TAC acute exercise	TAC chronic exercise	IL-6 acute exercise	IL-1ra acute exercise
*t* value	3.312	1.466	8.606	0.646	10.625	0.139	11.604	2.215	5.192	10.768
df	6	4	6	4	4	3	3	4	6	4
*P* value (1- tailed)	0.009	**0.108**	0.000	**0.276**	0.000	**0.448**	0.000	0.045	0.001	0.000
*P* value (2-tailed)	0.018	**0.216**	0.000	**0.553**	0.000	**0.897**	0.001	0.091	0.002	0.000

*GSH* reduced glutathione, *TBARS* thiobarbituric acid reactive substances, *PC* protein carbonyls, *SOD* superoxide dismutase, *TAC* total antioxidant capacity, *IL-6* interleukin-6, *IL-1ra* interleukin-1 receptor antagonist

Bold type indicates publication bias (i.e. *p* > 0.10)

## Discussion

Sports scientists and coaches are increasingly realising the value of redox homeostasis changes in athletes’ training adaptations. However, we have not yet been able to implement all these measurements efficiently and routinely within an applied sports science setting. There are many biomarkers, conditions, and protocols that make this implementation challenging for the majority of sports science “support” staff. Therefore, we aimed through this paper to use the following paragraphs (par. 4.1 to 4.6) to make it clearer to individuals that have an interest in the field and further understand results related to each variable. Additionally, we performed the meta-analysis to identify which markers are more sensitive and display significant variations and could, therefore, be useful to practitioners.

Based on the meta-analysis outcomes, significant alterations in oxidative stress and cytokines levels are noted after acute exercise, ranging from moderate to extremely large. In contrast, the variations after chronic exercise ranged from trivial to moderate (Table 5). However, we should be cautious with how those results are interpreted and whether supplementation can be provided, as recent studies suggest that some alterations may display the necessary signalling for performance adaptations [9, 39]. The results from the Downs and Black checklist of studies emphasised many areas in need of improvement to increase the quality of research in this field. Specifically, improvement is required mainly for internal validity—study bias, internal validity confounding—selection bias, and reporting of probability and important adverse outcomes. The high heterogeneity in specific meta-analysis advocates the need for further exploration and requiring consistency when we deal with the assessed variables. Many reasons can increase heterogeneity such as the study design, duration of follow-up of protocols, or the accuracy of results. Besides, the observed publication bias for specific variables (TBARS, GSH, and SOD) following long-term periods of training further supports those findings. In the next sections, analytical findings from a broad range of relevant studies in adolescent athletes are presented.

**Table 5:** The effect size (ES) of changes following acute or chronic exercise

Biomarker	Acute exercise	Chronic exercise
TBARS	Large increase (ES 1.317; *p* = 0.001)	Small decrease (ES 0.568; *p* = 0.077), n.s
GSH	Large decrease (ES -1.701; *p* = 0.001)	Moderate increase (ES 1.131; *p* = 0.005)
SOD	n/a	Trivial change (ES 0.513; *p* = 0.026)
PC	Extremely large increase (ES 4.164; *p* = 0.001)	n/a
TAC	Moderate increase (ES 1.057; *p* = 0.001)	Trivial change (ES 0.055; *p* = 0.756), n.s
IL-6	Very large increase (ES 2.291; *p* = 0.000)	n/a
IL-1ra	Large increase (ES 1.599; *p* = 0.012)	n/a

*GSH* reduced glutathione, *TBARS* thiobarbituric acid reactive substances, *PC* protein carbonyls, *SOD* superoxide dismutase, *TAC* total antioxidant capacity, *IL-6* interleukin-6, *IL-1ra* interleukin-1 receptor antagonist, *n.s* non-significant, *n/a* non-applicable

### Lipid Peroxidation

Meta-analysis outcomes confirmed that the ES of observed modifications in TBARS following a short period of training or performance testing is indeed large (ES 1.317; 95% CI 0.522 to 2.112). After a short duration event, TBARS tend to increase, provoking lipid peroxidation in adolescent athletes significantly. As the heterogeneity was high, the “between studies” variance in ES was estimated at *t*^2^ = 1.153. However, the use of TBARS for the monitoring of long-term changes needs further investigation as the effect size was small (ES 0.568; 95% CI − 0.061 to 1.197) and publication bias (PB) was noted. Many reasons could have contributed to PB, such as the use of different duration protocols and the small samples in the included researches.

#### Acute Exercise

In most published investigations, greater levels of lipid peroxidation have been mentioned after a short period of exercise or shortly after a performance assessment. However, a few studies found no noticeable changes [18, 34, 42, 47–49], and a diminished level of lipid peroxidation was reported in two studies [45, 52]. Increased TBARS or MDA concentrations have been revealed in young soccer players after a sequence of repeated sprints or a Loughborough Intermittent Shuttle Test [30, 49], in long-distance runners after culmination of a maximal test [26, 46], in rowers following a maximal simulated test [35], in swimming after performing different sets (i.e. interval or continuous) [33, 43, 58], and in handball players following a graded exercise test (GXT) [9]. In contradiction, in research where endurance athletes run the distance of a half marathon (21 km) twice throughout a training period (i.e. at the beginning and after 1 year of training; e.g. pre- and post-assessment), TBARS decreased immediately post-run in both efforts [45]. Further, TBARS decreased after a continuous aerobic exercise session in basketball but not after a high-intensity training session of the same extent [52]. Nonetheless, these markers remained unaltered in five studies where young basketball, handball, or healthy athletes were incorporated [42, 47–49, 51, 53].

#### Chronic Exercise

Based on the impact of prolonged training period on lipid peroxidation, heightened levels of TBARS were detected in junior athletes after 6 months of soccer practice [27] and in swimmers after the culmination of 13 weeks but not after 23 weeks of preparation [25]. Likewise, young track and field athletes had greater levels of TBARS after the execution of a maximal run test after 6 and 11 months of training, respectively. Those high rates remained elevated up to 1 h after the completed trial [26]. However, TBARS and MDA decreased in endurance runners when a 21-km race was repeated after 1 year of training, both pre- and post-evaluation [45], after 45 days of soccer training [53], and after 12 weeks of endurance exercise in soccer [54]. No deviations were noted after 23 weeks of swimming training [16].

### GSH Status

Based on the meta-analyses’ reports, the variations reported in swimming, rowing, handball, and athletics [9, 24, 35, 54] were considerable as they verified a large ES (ES − 1.701; 95% CI − 2.698 to − 0.705) suggesting the influence of those modalities on juvenile athletes. Also, the heterogeneity was high, and the “between studies” variance in ES was determined at *t*^2^ = 1.757. The effect size of those changes after a lengthier period was moderate and the publication bias significant.

#### Acute Exercise

The GSH level raised in 26 swimmers after interval or continuous swimming [33, 51]. In endurance runners, GSH levels only increased 4 h post-race, but no change was monitored at 2 h or 24 h post-test [44]. However, in a group of half-marathon runners, GSH decreased after a 21-km run during pre-season [45]. Similarly, GSH levels dropped in reply to a maximal test in trained adolescent handball players [9], after the completion of two distinctive swim tests (i.e. 100 m and 800 m) [32], after 12 sessions of 50 m intermittent swimming [24], and following a soccer practice [53]. Moreover, no variations were viewed in GSH levels in handball, where the competitors had to carry out a maximal test [47], and in rowing [35].

#### Chronic Exercise

An increased GSH level was detected in adolescent swimmers after 23 [16] or 13 weeks of exercising [25] and in junior soccer players during a 45-day training time [53]. A decreased GSH level was observed in an athletic population after a maximal test performed after 11 months of training [26] and after 6 months of regular soccer practice [27]. The overall ES of increment was moderate (ES 1.131; 95% CI 0.350 to 1.913). No shifts in GSH levels were discovered in athletics where long-distance runners completed a half-marathon run post-season [45]. However, long-term variations documented in swimming following short- or long-term periods of practice [25, 32] were more profound compared to other studies. It seems that athletes adapt to their antioxidant adaptations after longer periods of training.

### Endogenous Enzymatic Antioxidants

Meta-analysis was implemented for variations in SOD accumulation after a prolonged period of training since a minimum of five investigations (i.e. consisting of results feasibly to convert in common units) could be collected only for this condition. The ES of alterations in SOD was small (ES 0.581; 95% CI − 0.259 to 1.420) following long-term periods of work out and the publication bias significant. Besides, the between studies variation was found at *t*^2^ = 0.163.

#### Acute Exercise

Following short-term exercise, the levels of SOD reduced in three studies. SOD decreased in handball, where a maximal test and an ordinary exercise session were included, and in athletics [9, 45]. However, no difference was demonstrated after a GXT [9] or a 21-km half marathon race at the start of the period [45]. No fluctuations in SOD concentrations were reported after a treadmill test in athletics [46], handball [9], or endurance runners [44]. Yet, SOD was higher following a run consisting of ten sets of 2 × 200 m sprints in soccer players [30]. GPx was similarly high after a treadmill assessment completed by an athletic population [46] and after the execution of two different swimming trials (100 and 800 m) in juveniles [32]. CAT levels increased in a group of elite Greek rowers [35], in a team of female basketball players after their continuous aerobic exercise [52], and in adolescent track and field athletes shortly after the exercise period in response to a GXT (at the onset of the season) and soon after the testing [26]. However, such enzymes remained unaffected after a 21-km run at pre-evaluation trial [45]. Only one investigation presented a reduction in CAT levels [9].

#### Chronic Exercise

Both enzymes, SOD and CAT, were elevated after 6 months of soccer training [27] and 16 weeks of swimming training [25]. Moreover, in the swimming study, GPx substantially reduced [25]. However, CAT rose after 1 year of exercise and completion of a 21-km half marathon run [9], and immediately after a maximum trial performed at the end of an 11-month training season [26]. Besides, it persisted unchanged after 23 weeks of preparation in swimming [16]. Besides, SOD and GPx were unaltered during the practice season in soccer [36] but dropped after 1 month of training in soccer and in a group of endurance athletes who completed a 21-km run after 1 year of training [45, 54].

### Protein Modification

Likewise, only one meta-analysis was conducted for differences in PC concentration after a short period of training, since we could not gather more studies evaluating long-term modifications in juvenile athletes. The overall ES for alterations regarding PC were extremely large (ES 4.164; 95% CI 1.716 to 6.613) and the variance between the studies high (*t*^2^ = 8.718). Therefore, more consistency is recommended when the evaluation of PC is incorporated in studies.

#### Acute Exercise

PC increased after a sprint exercise in soccer, 2000 m maximal testing on a rowing ergometer [30, 35], and a short period of interval swimming [24]. They were also considerably above resting values immediately post and 1 h post-exercise in all athletics groups (athletes and controls) during pre-training testing [26]. However, carbonyls remained unaffected in two surveys in swimming [33, 43] and wrestling [50].

#### Chronic Exercise

PC rose noticeably after 16 weeks of swimming and after 11 months of athletic preparation [25, 26]. In the research mentioned above [26], PC levels remained substantially above resting values immediately post and 1 h post-exercise in both groups of young participants (trained adolescent and controls) at mid- and post-season screenings (except 1 h post- at mid-training) [26]. No meta-analysis was performed as the number of studies was limited.

### TAC Capacity

Although no publication bias was detected for studies related to the measurement of TAC in both conditions (i.e. acute and chronic), the ES of changes were moderate after a short period (ES 1.057; 95% CI − 0.044 to 2.158) but trivial following more extended periods of training. Also, the variance between the studies was low (*t*^2^ = 0.096 and 0.119, respectively).

### Acute Exercise

Most studies have shown a rise in the TAC of athletes at the end of discrete types of short-term exercise. Notably, an increase in TAC was noted following a series of sprint trials or a Wingate test in soccer [30, 31], a short interval swim of 12 × 50 m at an intensity between 70 and 75% [24], 2 sets of 4 × 50 m swimming [51], and a simulated rowing ergometer test [35]. Furthermore, TAC increased immediately at the end of the exercise in all groups in track and field (young athletes and controls) conducted at baseline testing [26]. In comparison, the antioxidant capacity declined in basketball after the completion of a circuit program [34], in handball after a maximal trial on the bike [9], and after a Loughborough Intermittent Shuttle Test in soccer players [49]. The total antioxidant capacity (T-AOC) remained stable following a 21-km race, at the beginning and end of a practice period [8], and regular soccer training [53].

#### Chronic Exercise

Long-term exercise was not found to result in considerable variations in TAC in most studies. These considerations had been recorded in several sports, such as after 16 and 23 weeks of swimming training [16, 25] and after 1 month of soccer practice [53]. However, no significant discrepancies were present after a repeated 21-km run at the end of the training course [45]. In contrast, increments in TAC were listed immediately after and 1 h after a maximal experiment was performed at mid- and post-season in two groups (trained adolescents and controls) in athletics [26].

### Inflammatory Mediators

Almost in all considered studies, IL-6 and IL-1ra levels significantly increased after the performance of a short duration of exercise with the ES being very large (ES 2.291; 95% CI 1.082 to 3.501) and large (ES 1.599; 95% CI 0.347 to 2.851), respectively. The between studies variance was high for both cytokines, i.e. (*t*^2^ = 2.621 and 2.107, respectively). More specifically, IL-6 was raised in basketball players during the competitive period compared to the preparatory period [55]. In volleyball players, this cytokine increased either after a standard practice or after 7 weeks of training [28, 29, 58]. Likewise, excessive IL-6 levels have been confirmed in other team sports such as water polo (after 90 min of formal training) and handball (after a course of different run drills) [37, 39]. Moreover, in the cross-country, female adolescent runners demonstrated high IL-6 levels following typical endurance training [41]. The results observed in wrestling were similar either after one wrestling training session or following half of a school’s training period [38, 40].

In all the examined studies related to volleyball, IL-1ra was unaltered [28, 29, 58]. Unaffected IL-1ra concentration was also observed in handball after the performance of a series of 250 m run sprints [37]. Considering long-term wrestling training, IL-1ra also increased at the mid-year screening during a school year [40]. The long-term exercise enhanced the TNF-α level in basketball players during competition [55], in wrestling after 90 min of exercise [38], and in tennis immediately after the tournament [57]. Nonetheless, no marked variations in TNF-α were noted when wrestling practice was implemented for one training season [40] or in female water polo players after the culmination of 90 min of training [39]. The anti-inflammatory cytokine IL-10 was measured in three surveys. IL-10 level was reduced in competitive basketball season [55] and reached its peak after 3 and 14 days of tennis camp [26] but continued to be stable after regular training in handball, which consisted of a set of 4 × 250 m runs on a treadmill [37]. Meta-analysis was performed for IL-6 and IL-1ra. It was found that the ES of shifts in IL-6 and IL-1ra were very large following various training modalities such as volleyball, wrestling, water polo, cross-country, and handball.

### Strengths and Limitations of the Review

Throughout our investigation about how specific modalities and periods of exercise can affect the redox and inflammation status of youth athletes, several issues were identified. The most remarkable was the use of various biomarkers to define the same type of oxidative stress damage or body’s antioxidant capacity, which mostly represent differing end-products. Additionally, the use of diverse analytic methods, applied testing protocols, and the expression of results in different units was among the limitations. Therefore, by employing precise criteria, we aimed to carry out the current meta-analyses and generalise results about variations on redox and inflammation status of young athletes.

## Conclusions

The results of the present review demonstrated that both short- and long-term exercise are evident stressors of the athletes’ redox status leading to acute and chronic responses, respectively. Alterations in redox homeostasis biomarkers following acute exercise are more significant, while the variations after chronic exercise ranged from trivial to moderate. However, we should be cautious with how those results are interpreted. Of the inflammatory mediators, IL-6 and IL-1ra were the most sensitive cytokines to training paradigms in young athletes, whereas other cytokines such as TNF-α and IL-10’s sensitivity depended on the type of sport. In addition, the publication bias and high heterogeneity in specific meta-analyses advocate the need for further exploration and consistency when dealing with the assessed variables.

## Supplementary information

**Additional file 1.** Appendix 1. PRISMA Check list. Appendix 2. The results of the methodological quality assessment using a modified 26-item Downs and Black checklist for all studies in meta-analysis.

## Data Availability

All data generated or analysed during this study are included in this published article [and its supplementary information files].

## Abbreviations

RONS: Reactive oxygen and nitrogen species
SOD: Superoxide dismutase
GPx: Glutathione peroxidase
CAT: Catalase
TBARS: Thiobarbituric acid reactive substances
MDA: Malondialdehyde
LOOH: Lipid hydroperoxides
GSH: Reduced glutathione
PC: Protein carbonyls
TAC: Total antioxidant capacity
IL-6: Interleukin-6
IL-1ra: Interleukin-1 receptor antagonist
TNF-α: Tumour necrosis factor-α
IL-10: Interleukin-10
