# Supplementary material for: Redox Homeostasis and Inflammation Responses to Training in Adolescent Athletes: a Systematic Review and Meta-analysis
Source: Sports Med Open. 2020 Aug 3;6:34. doi: 10.1186/s40798-020-00262-x (PMC7399016; doi:10.1186/s40798-020-00262-x)
Supplement: Supplementary file 1 — Additional file 1. Appendix 1. PRISMA Check list. Appendix 2. The results of the methodological quality assessment using a modified 26-item Downs and Black checklist for all studies in meta-analysis. [file 40798_2020_262_MOESM1_ESM.docx]

**Appendix 1.** PRISMA Check list.


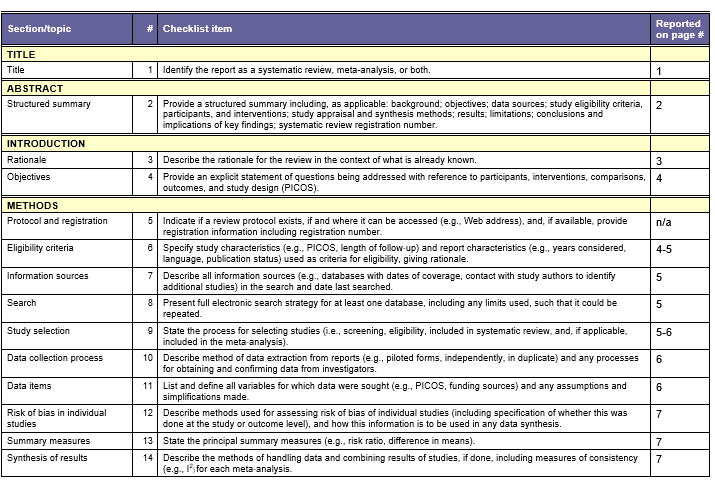


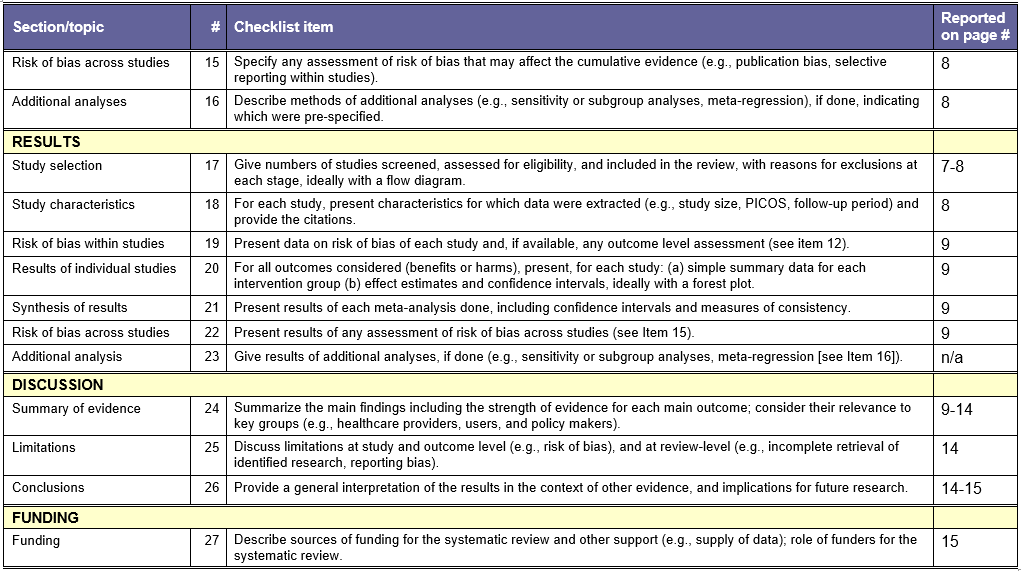


**Appendix 2.** The results of the methodological quality assessment using a modified 26-item Downs and Black checklist for all studies in meta-analysis


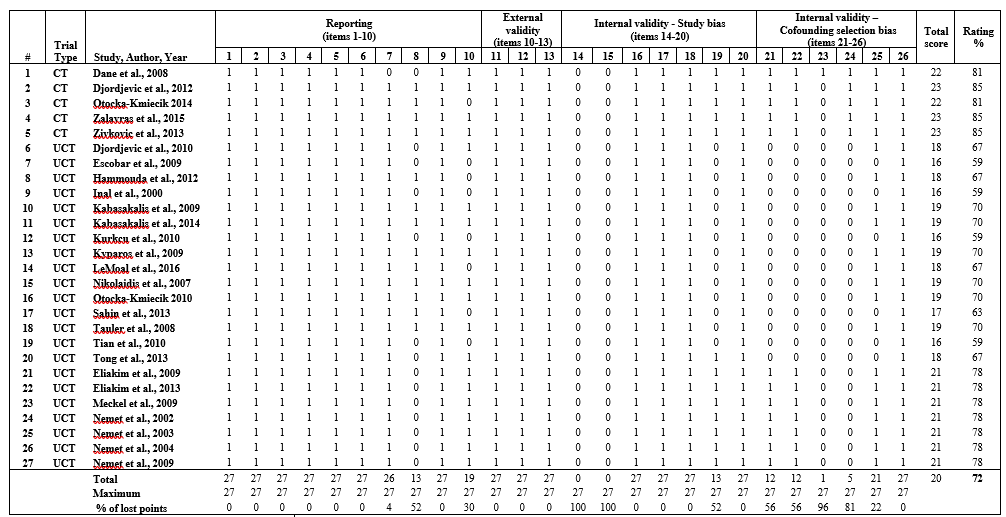


1= criteria was met, 0 = criteria was not met
